# An Application of Item Response Theory to Scoring Patient Safety Culture Survey Data

**DOI:** 10.3390/ijerph17030854

**Published:** 2020-01-29

**Authors:** Heon-Jae Jeong, Hsun-Hsiang Liao, Su Ha Han, Wui-Chiang Lee

**Affiliations:** 1 The Care Quality Research Group, 174 Toegye, Chuncheon, Gangwon 24450, Korea; hj9571@gmail.com; 2 Joint Commission of Taiwan, No. 31, Sec. 2, Sanmin Rd., Banqiao Dist., New Taipei City 220, Taiwan; shing@jct.org.tw; 3 Department of Nursing, Soon Chun Hyang University, 31 Soon Chun Hyang 6-gil, Dongnam-gu, Cheonan-si, Chungcheongnam-do 31151, Korea; jasmin720@sch.ac.kr; 4 Department of Medical Affairs and Planning, Taipei Veterans General Hospital, No. 201, Sec. 2, Shipai Rd., Beitou Dist., Taipei City 112, Taiwan; 5 National Yang-Ming University School of Medicine, Institute of Hospital and Healthcare Administration, No. 155, Sec. 2, Linong St., Beitou Dist., Taipei City 112, Taiwan

**Keywords:** item response theory, patient safety, questionnaire, safety culture, survey, teamwork

## Abstract

Patient safety culture is important in preventing medical errors. Thus, many instruments have been developed to measure it. Yet, few studies focus on the data processing step. This study, by analyzing the Chinese version of the Safety Attitudes Questionnaire dataset that contained 37,163 questionnaires collected in Taiwan, found critical issues related to the currently used mean scoring method: The instrument, like other popular ones, uses a 5-point Likert scale, and because it is an ordinal scale, the mean scores cannot be calculated. Instead, Item Response Theory (IRT) was applied. The construct validity was satisfactory and the item properties of the instrument were estimated from confirmatory factor analysis. The IRT-based domain scores and mean domain scores of each respondent were estimated and compared. As for resolution, the mean approach yielded only around 20 unique values on a 0 to 100 scale for each domain; the IRT method yielded at least 440 unique values. Meanwhile, IRT scores ranged widely at each unique mean score, meaning that the precision of the mean approach was less reliable. The theoretical soundness and empirical strength of IRT suggest that healthcare institutions should adopt IRT as a new scoring method, which is the core step of processing collected data.

## 1. Introduction

Since the end of the 20th century, when the landmark report “To err is human: Building a safer system” was released, patient safety has been considered to be one of the most important topics in healthcare [1,2]. Thus far, many resources have been invested in improving safety globally [2,3]. The experience from such efforts makes it clear that the success of any endeavors to create errorless care is largely affected by the level of the safety culture of the target place [4,5,6,7,8,9]. Therefore, understanding the precise situation of a safety culture is considered a vital component in ensuring the success of safety programs, and many survey instruments have been developed and applied in the various areas of healthcare [8,10,11,12,13,14]. The usefulness of such instruments in improving safety per se is undeniable, and a large amount of data have been collected. However, few studies have looked into improving the methods of processing the collected data; such ignorance might have lessened the effectiveness of the instruments.

Most survey questionnaires use a Likert scale for response options of items. To illustrate, the Safety Attitudes Questionnaire (SAQ), one of the most popular instruments [13,14], uses a 5-point Likert scale (1 = disagree strongly, 2 = disagree slightly, 3 = neutral, 4 = agree slightly, 5 = agree strongly) to measure respondents’ attitudes toward items such as “Staff input is well received in this clinical area” [15]. However, a Likert scale is an ordinal (i.e., ordered categorical) scale by definition, where the difference between any two adjacent response options is not necessarily the same difference as between another pair of adjacent options [16]. Thus, we cannot be sure in stating that the difference between “disagree strongly” and “disagree slightly” is the same as between “agree slightly” and “agree strongly.” In addition, these differences vary across items. Therefore, unlike an interval scale built with the same intervals on a trait continuum, a Likert scale does not allow us to calculate the traditional mean score of items [17]. Although we relax such restrictions arising from this theoretical concept about scale, a practical issue remains: When the number of items is small in a construct (domain), the mean, or summed score, does not offer sufficient resolution to detect subtle changes or differences in the safety culture level.

Item response theory (IRT) provides a practical solution to these problems. It estimates each item’s properties, such as where on the safety culture continuum respondents switch their responses to the adjacent option. Fayers defined IRT as “a model-based measurement, also known as latent trait theory, in which trait level estimates depend on both a person’s responses and on the properties of the items that were administered” [18]. This definition implies that, once items are calibrated and their properties are revealed, the safety culture of respondents can be estimated at a highly reliable precision by appropriately handling responses on a Likert scale [19].

We applied IRT to the Chinese version of SAQ (SAQ-C), which has been used in Taiwan for the past decade. During the development of the SAQ-C, the stress recognition (SR) domain from the original version developed by Dr. Bryan Sexton was excluded, because it did not perform well in Taiwan. This issue was not Taiwan or SAQ-C specific. Several countries that adopted the SAQ reported the same problem [20]. In addition, two items in double negative sentences from the original version did not function well when translated into Chinese; thus, they were removed. This was not an SAQ-C specific issue either [21]. Eventually, the remaining 30 items on the SAQ-C contained five domains: teamwork climate (TC: 5 items), safety climate (SC: 6 items), job satisfaction (JS: 5 items), perception of management (PM: 10 items) and working conditions (WC: 4 items) [15,22].

This study tried to examine whether the IRT approach is suitable for scoring already collected SAQ-C responses, and determine whether IRT can produce scores in higher resolution than the traditional mean approach. By examining the differences between the two scoring methods for individual responses, while holding the other steps constant across the methods, we intended to answer whether it is worth considering a switch to the new methods.

## 2. Methods

### 2.1. Data Source

We used SAQ-C data collected from 200 hospitals in Taiwan from 31 May to 30 June,2008, the first national administration of the survey. Item parameters theoretically do not vary across respondents, but if a person takes the same survey multiple times, the parameters might change gradually [23]. Considering the voluntary and anonymous nature of SAQ-C administration in Taiwan, such item parameter drift (IPD) cannot be traced, and the proportion of drifters is unknown. Thus, using the first administration dataset was the most reasonable option for item calibration to avoid the risk of IPD.

### 2.2. Calculating Traditional Mean Domain Scores

The suggestions from the rubric provided by the developer of the SAQ were used to obtain a mean score of each of the five domains: 1 is subtracted from each of the raw item scores (1 to 5), and then multiplied by 25. The average of the converted item scores is the mean domain score on a scale of 0 to 100.

### 2.3. Confirmatory Factor Analysis with IRT

The original SAQ and SAQ-C were validated using confirmatory factor analysis (CFA), based on the assumption that the responses were on an interval scale (continuous variable) [22]. Therefore, we first proved that IRT, the paradigm that treats responses as an ordinal categorical variable, can be applied to our dataset. We developed a correlated factor model to conduct CFA, in order to maintain consistency with the model used when the SAQ-C was developed. Specifically, we used a graded response model (GRM) of IRT, because the response options were polytomous [24]. Since it is broadly acknowledged that hospital levels, such as medical centers and regional, district, and psychiatric hospitals (Joint Commission Taiwan classification) may influence culture level to some degree [25], a multigroup component was added to the CFA model. Eventually, CFA using a multigroup- multidimensional-IRT (MIRT) model was built, and both goodness-of-fit indices and item properties were achieved.

### 2.4. Calculating IRT-Based Domain Scores

After the model fit was established, we ran a unidimensional IRT model with expected a posteriori (EAP) computation method for each domain, and estimated IRT scores in standard deviation (SD) as a unit of reporting [26]. Note that, unlike SD in classical test theory (CTT), where it can be used to locate a person’s level within a certain group, SD in IRT is less group-specific, and thus, fewer issues are expected in a group-to-group comparison for longitudinal tracking of domain scores [19]. Achieved IRT scores were rounded to 2 decimal places for practical purposes.

### 2.5. Comparing Traditional Mean and IRT-Based Scores

To examine the resolution of the scores obtained from each of the two methods, we used the number of unique domain scores. Theoretically, the TC domain can possess 21 unique values because it consists of five items measured by a 5-point Likert scale ((4 × number of items) + 1). In the same way, SC, JS, PM and WC domains can have up to 25, 21, 41 and 17 unique scores, respectively. We counted the actual number of unique mean scores by domain from the data. The number of unique IRT scores was also counted by domain. Then, we observed how widely EAP scores varied at each of the unique mean domain scores. The correlation coefficient for the scores from the two methods was also calculated for each domain.

Analyses were performed using the statistical software packages flexMIRT 3.51 (Vector Psychometric Group, LLC, Chapel Hill, North Carolina) and Stata 16.1 (Stata Corp., College Station, Texas). This study was approved by the Institutional Review Board of the Taipei Veterans General Hospital, Taiwan (2017-07-015CC#1).

## 3. Results

### 3.1. Characteristics of Respondents

The dataset contained 45,242 questionnaires. Among them, we excluded 8089 questionnaires for two reasons. First, since each domain of the SAQ consists of a small number of items, common techniques to treat missing values, such as calculating the mean score from only the answered items or multiple imputation, could not be used. Even if they were applied, such ad hoc approaches might have prevented the summed score to EAP conversion described in a later section. Second, because we conducted a multi-group analysis based on the hospital level, we unavoidably excluded questionnaires with the hospital level variable left unanswered. Eventually, 37,163 questionnaires were analyzed. As described in Table 1, seemingly unbalanced distributions were observed in most categories, such as the fact that 88.2% were female, 55.5% were in their 20s, and 70.6% were nurses. There were 20 medical centers in Taiwan, but they made up a 44.7% share of collected questionnaires. Although such imbalance may reflect the reality of Taiwan’s healthcare topography, sensu stricto, there was an unavoidable risk of bias arising from survey administration that was conducted in an anonymous manner. We could only say that as the first and the most extensive dataset collected across the country, it was relatively stable and free from the risk of IPD. Before moving on, we should make what follows clear. Some researchers have argued that the representativeness of the dataset is not too important “because the IRT paradigm is relatively generous to sample characteristics.” [27] However, no studies can use such an argument as protection against all criticism about sample representativeness issues, and this study is no exception.

### 3.2. Results of Confirmatory Factor Analysis

**Figure 1:**
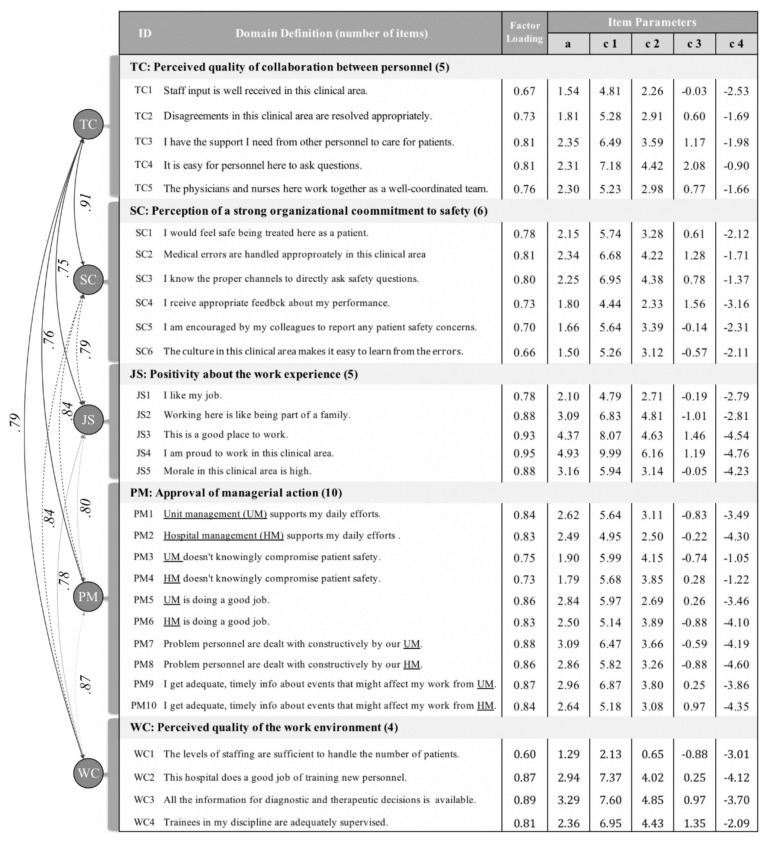
Item and model parameters. Note: In the multidimensional IRT (Item Response Theory) model, the intercepts are preferred over ‘b,’ the difficulty parameter that is dominantly used in the unidimensional model. (TC: Teamwork Climate; SC: Safety Climate; JS: Job Satisfaction; PM: Perception of Management; WC: Working Climate).

In the middle column of Figure 1 lie the factor loadings of each item, which were all satisfactory: 0.60 (WC1)–0.95 (JS4) [28]. There were correlation coefficients between domains on the left of the table, and all of them were high, spanning from 0.75 between TC and JS, to 0.91 between TC and SC. As for fit indices of the model as a whole, the full-information goodness-of-fit (GOF) tests based on popular Pearson’s χ2 or G2-based statistics were not reliable because the contingency table from this 30-item ordinal scale dataset was too sparse. Therefore, we used limited-information GOF tests instead, with M2* statistics [29,30], the root mean square error of approximation (RMSEA) and the non-normed fit index (NNFI; also known as the Tucker-Lewis index). Both showed satisfactory results: 0.03 for RMSEA (cut-off <0.06) and 0.98 for NNFI (cut-off >0.95) [31]. Note that, the repertoire of limited-information statistics is small compared to that of full-information statistics at the moment of writing this [30].

### 3.3. Item Properties

The five columns on the right side of Figure 1 describe the SAQ-C item parameters, ‘a’ to ‘c4,’ calibrated from CFA using an MIRT. Furthermore, ‘c1–c4’ are called intercepts on the attitudinal level, at which respondents switch to the adjacent response options (e.g., c1 is the intercept between “disagree strongly” and “disagree slightly”). The value of intercepts is inversely proportional to the location of the switching points. Meanwhile, ‘a’ is the discrimination parameter implying how sharply a respondent switches her answers at a location on the safety culture continuum denoted by the intercepts, ‘c1’ to ‘c4.’ In each domain, the values of intercepts, ‘c1–c4’, and discrimination parameter, ‘a’ varied significantly by item. To illustrate, for TC1 and TC4, ‘a’ was 1.54 and 2.31, respectively, and ‘c1’ was 4.81 and 7.18, respectively. The parameters suggested that items performed quite differently, and IRT’s weighted score might better reflect the reality. Although not included in the table due to space constraints, standard errors were obtained and the majority were 0.01 with others of 0.02.

### 3.4. Comparing the Results from Two Scoring Methods

After confirming that the model fit suffices from the correlated factor model, we estimated IRT scores by domain using a unidimensional IRT model. To conserve space, we do not describe individual item parameters, but the pattern was similar to that of the multidimensional model shown above. As described in Table 2, the traditional mean scoring scheme yielded its theoretically maximum unique values: 21, 25, 21, 41, and 17 for TC, SC, JS, PM, and WC, respectively. On the other hand, IRT provided us 473, 498, 464, 511, and 440 unique scores for each of the five domains, respectively, suggesting that scores were achieved at a much higher resolution with IRT. By increasing the number of decimal places of EAP scores, we can obtain an even higher resolution. For all five domains, the traditional mean scores spanned from 0 to 100 (the range was 100), meaning that there were respondents who gave “disagree strongly” to all items, and also respondents who answered “agree strongly” to all items. The lowest values of the IRT domain scores varied between −3.43 of TC and −2.88 of WC, while the highest values spread between 1.83 of TC and 2.37 of PM. Therefore, the ranges of scores in each domain spanned from 5.03 in WC to 5.74 in PM. Note that, unlike the range in scores from the traditional mean (100 for all domains), the range of scores from IRT varied by domain, as expected.

**Figure 2:**
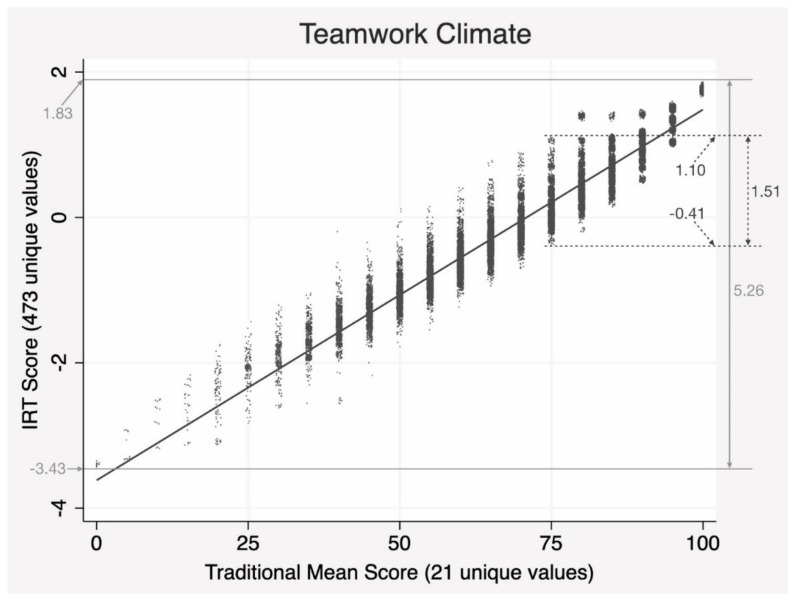
A visual manifestation of the relationship between scoring methods. Note: dots are jittered slightly for a better view.

The correlation coefficient between the two methods should be viewed with caution. It spanned from 0.96 in WC to 0.99 in PM. Such a high correlation coefficient certainly means a strong linear relationship, but it should not be interpreted as a single line-like relationship. See Figure 2. Although the linearity did exist, IRT scores for each unique score achieved from the traditional mean approach varied widely. To illustrate, we focused on a single value of 75 from the traditional mean scoring; the value which the original SAQ rubric regards as the threshold distinguishing between respondents with and without good safety attitudes. There, the range of IRT scores was at 1.51 (between −0.41 and 1.10). It took up as much as 28.7% of the total range of TC scores (1.51/5.26). This phenomenon raised questions about the precision of the traditional scoring scheme, where item-specific properties based on the ordinal categorical characteristics of a Likert scale are not taken into account.

## 4. Discussion

### 4.1. Which Method Is Correct or Better?

Scaling is the process that assigns a value to a respondent according to the respondent’s position on the safety attitudes continuum [32]. To calculate a mean domain score, a strong assumption should hold: all items in a domain should be built on an interval scale (or ratio scale), and they should be parallel instruments. That is if a respondent answered 2, 2, 3, 4, 5 to the five consecutive items in TC, and another respondent answered 5, 4, 3, 2, 2, the mean TC scores of the two people should be the same. However, as shown in Figure 2, items are not parallel: each unique mean score corresponded to a very wide range of scores achieved from IRT. It is clear that responses on a Likert 5-point scale in the SAQ should be scored using IRT that can relax the parallel item assumption, and treat response options on an ordinal scale, as it should. The results suggested that IRT provides much higher precision with greater resolution than the traditional means [33,34].

### 4.2. Barriers against Switching to the IRT Methods

Understanding IRT might overwhelm those who want to switch. Indeed, IRT is a complex area, and it is natural to feel uncomfortable with what one does not understand thoroughly. We recommend reviewing an article by Jeong et al. (2016). Although it was based on a small pilot study, the authors depicted most of the fundamental concepts of IRT visually using real SAQ data (Korean version) [35].

Meanwhile, the scarcity of computing resources has also been a critical issue. It is commonly assumed that if personnel in charge of processing SAQ data wanted to apply IRT, they would have to deal with hours of waiting for the results coming out from a computer. However, this is because of a significant misunderstanding. Applying IRT to achieving SAQ scores consists of two steps: calibrating items and calculating scores using the item parameters from the calibration step. The majority of the time is spent on calibrating items. Once the parameters are revealed, they can and should be used for scoring future data without recalibration. This is a theoretically sound way of scoring, and also buttresses the longitudinal comparison of scores. Such scoring only processes can be done quickly. Furthermore, if one wants to obtain only domain scores, then a unidimensional model is enough, where both the calibrating and scoring steps can be done in a short time. In addition, we used Bock and Aitkin’s Expectation-Maximization algorithm because it provides GOF indices from CFA. If one does not need GOF tests, a much more efficient Metropolis-Hastings Robbins-Monro (MH-RM) algorithm would be a good alternative. MH-RM is a ‘Markov chain Monte Carlo-based sampler’-driven data-augmented approximation that can easily handle complex models with a large dataset at a dramatically increased speed [36,37].

Even when survey responses from the past administrations have already been scored in the form of traditional mean, and the raw data were redacted, there is still a solution to make a switch to IRT. By using the EAP score, we can generate a conversion table from the traditional mean (or summed score) to the IRT score. Such backward compatibility was one of the reasons why we chose EAP over the other scoring methods: Most IRT software tools at any budget level provide the summed score to the EAP conversion table. Although such conversion inevitably coveys a considerable amount of variance, Jeong et al. (2017) pointed out that if the interests lie in a group mean and not an individual respondent’s score, the converted IRT scores performed well without raising significant issues. When backward compatibility of scores does matter, the rule of thumb is to calibrate the earliest dataset that contains the raw form of data. Then, to any dataset not in raw form, we apply the score conversion methods. Of course, for a dataset with raw data, we can calculate EAP scores by using the calibrated item parameters. In addition, the EAP calculation is known to have relative strength compared to the other scoring methods in the longitudinal analysis that track changes in SAQ scores [26].

Understanding the IRT scores in SD may confuse users who are accustomed to the 0–100 scale and may find difficulty in interpreting scores in SD. They might get lost, especially when attempting to obtain the percent agreement (PA). However, this should not be an excuse for rejecting the adoption of IRT in the SAQ: We can linear transform the IRT scores in standard SD to match the traditional SAQ scoring format, ranging from 0 to 100 at any time [38]. To the transformed score, one can apply any analysis methods as they used to do to the traditional mean score.

## 5. Conclusions

Using a large national dataset, this study proves that IRT can successfully be applied to the SAQ-C. There were clear strengths in using IRT, such as treating SAQ response options as an ordinal scale, and obtaining very high precision and resolution of achieved scores. With all due respect, by providing practical solutions to overcome the barriers that have hampered healthcare from switching to the IRT paradigm, we suggest that quality and safety personnel consider adopting the new IRT-based scoring method of collected data, or at least use IRT to check the reliability of traditional mean score approaches.

## Figures and Tables

**Table 1:** Characteristics of respondents.

Characteristics	N	%
Gender		
Male	4,375	11.8
Female	32,788	88.2
Age group (years)		
≤20	110	0.3
21–30	19,668	55.5
31–40	11,656	31.7
41–50	4,422	12.0
51–60	829	2.3
>60	58	0.2
Job types		
Physicians	2,369	6.4
Nurses	26,229	70.6
Technicians	3,054	8.2
Pharmacists	1,835	4.9
Administrative staff	792	2.1
Others	806	2.2
Missing	2,078	5.6
Hospital levels (N)		
Medical centers (20)	16,613	44.7
Regional hospitals (57)	13,510	36.4
District hospitals (104)	5,698	15.3
Psychiatric hospitals (19)	1,342	3.6
Total	37,163	100

**Table 2:** Comparison between traditional mean and IRT scores. (TC: teamwork climate, SC: safety climate, JS: job satisfaction, PM: perception of management, WC: working condition, IRT: item response theory).

Domain	N		Range of Scores					Correlation Between Methods
			Mean		IRT			
	Mean	IRT	Lowest	Highest	Lowest (a)	Highest (b)	|a–b|	
TC	21	473	0	100	−3.43	1.83	5.26	0.97
SC	25	498	0	100	−3.41	2.06	5.47	0.97
JS	21	464	0	100	−3.15	1.95	5.10	0.97
PM	41	511	0	100	−3.37	2.37	5.74	0.99
WC	17	440	0	100	−2.88	2.15	5.03	0.96
